# Estimating the regional distribution of men who have sex with men (MSM) based on Internet surveys

**DOI:** 10.1186/1471-2458-9-180

**Published:** 2009-06-11

**Authors:** Ulrich Marcus, Axel J Schmidt, Osamah Hamouda, Michael Bochow

**Affiliations:** 1 Robert Koch Institute, Dept. Infectious Diseases Epidemiology, Post box 650261, 13302 Berlin, Germany; 2 Social Science Research Center Berlin (WZB), Public Health Unit, Reichpietschufer 50, 10785 Berlin, Germany

## Abstract

**Background:**

Measurement of prevalence and incidence of infections in a hard to reach population like men who have sex with men (MSM) is hampered by its unknown size and regional distribution. Population-based surveys have recently been used to estimate the total number of MSM, but these surveys are usually not large enough to measure regional differences in the proportion of MSM in the population. We explored the use of the proportional regional distribution of participants of large internet-based surveys among MSM from Germany to estimate the regional distribution of MSM in Germany.

**Methods:**

We compared participants from two separate MSM behavioural surveys with each other and with the distribution of user profiles of the largest contact and dating website for gay and other MSM in Germany in terms of the representativeness of the regional distribution. In addition, we compared the regional distribution of reportedly HIV positive survey participants with the regional distribution of HIV notifications within the national surveillance system that can be attributed to transmission through homosexual contacts.

**Results:**

Regional distribution of survey participants was almost identical in both surveys, despite little overlap between survey participants. Slight discrepancies between surveys and user profiles could be observed. Proportional regional distribution of survey participants with HIV diagnosis resembled national surveillance data.

**Conclusion:**

Considering the difficulties to obtain representative data by other sampling methods for "hidden" populations like MSM, internet-based surveys may provide an easy and low cost tool to estimate the regional population distribution – at least in Western post-industrialized countries. Some uncertainties remain about the exact place of residence of MSM in larger cities or catchment areas of these cities. Slightly different results from different datasets may be due to unequal popularity of MSM websites in different regions. The total population size of the MSM population can be estimated based on e.g. data from representative national population surveys. Both estimates can then be combined to calculate the absolute size of regional MSM populations.

## Background

National infectious disease surveillance data for sexually transmitted infections, if they collect information on the gender of sexual partners, usually refer diagnosis or infection incidence in MSM to the general population or the general male population. However, this approach neglects specific migration processes that lead to a concentration of sexual minorities such as men who have sex with men in larger cities, especially if they self-identify as gay.

The measurement of prevalence and incidence of health conditions or infections in subpopulations such as men who have sex with men (MSM) is hampered by the unknown size and regional distribution of this "hidden" population. Problems arise from the definition of MSM, the identification of MSM in population based surveys, and the unequal distribution of MSM in the general population, which would require very large sample sizes for general population surveys to establish a valid pattern of the regional distribution of MSM [1].

Previous efforts to estimate subpopulation-specific incidence and prevalence of HIV therefore often remained restricted to metropolitan areas with higher proportions of MSM in the population [2]. Some approaches rely on a multitude of local and regional studies with differing methodologies [3], and some use samples of gay men recruited in health care institutions or community venues, which may contain numerous biases and are not representative for MSM.

Other methods that have been explored to estimate regional distribution of MSM are the use of markers like clustering of registered civil partnerships of same sex couples in national census data and participation in telephone surveys for MSM [4-7].

Population-based surveys have been used to explore the percentage of MSM in the general population [8,9], but the validity of such estimates relies on the acceptance of talking about same sex experience. Fortunately, it seems that in Western post-industrialized countries, asking people about their sexual preferences is becoming more and more acceptable [8,10]. It has also been reported that in computer-assisted interviews respondents are more willing to answer sensitive questions, such as on sexual preferences, than in person-to-person interviews [11]. MSM in population based surveys are usually identified by self-reported sexual contact with other men, either during lifetime or during more recent time periods. Men who are ambiguous about their desire to have sex with other men, who are sexually abstinent or who have predominantly heterosexual contacts will only partly self identify as MSM in such surveys, and the willingness to report homosexual contacts or sexual preferences depends on the wording of questions, survey methods and settings [1]. Some level of underreporting of stigmatized behaviour will therefore inevitably persist, and the proportion of the male population that can be identified as MSM in surveys is strongly depending on the social acceptance of same sex sexual relations and may thus change over time and differ between countries and regions. However, the data derived from such surveys can still be quite useful to estimate the "identifiable" size of a population at risk for sexually transmitted infections (STIs), because men who are reluctant to report sexual experiences with other men in the relatively anonymous context of a survey, may even more rarely report homosexual contacts to a physician if diagnosed positive for HIV or other STIs.

### Objectives

We hypothesize that due to the rapid adaptation of online chatrooms and other internet contact sites among MSM, the regional distribution of participants of larger MSM internet surveys can be used to estimate the regional distribution of the whole "identifiable" MSM population, at least in countries where communication infrastructure allows wide spread and equal internet access in all regions.

To verify this hypothesis we explored the consistency of the regional distribution of participants of two separate MSM behavioural surveys and compared these distributions with one of the largest available dataset on MSM: the user profiles of Germany's leading MSM dating and contact site, called GayRomeo ™. Nearly 300,000 user profiles (as of early 2008) can be sorted by federal state and 'city' – which is usually the next bigger city with more than 100,000 inhabitants.

In order to validate our findings with a data source that is independent of internet access, internet use, or study participation (and therefore lacks participation bias), we compared regional distribution of HIV notifications (attributed to homosexual contacts) in the national infectious disease surveillance system with the regional distribution of survey participants who reported to be HIV positive. The underlying argument is that if the regional distribution of a subgroup – MSM with a diagnosis of HIV – is representative, then the regional distribution of the whole convenience sample is likely to be representative as well.

## Methods

### Data on MSM from behavioural surveys

**The KABaSTI-study **(Knowledge, Attitudes and Behaviour as to Sexually Transmitted Infections) was a behavioural survey among MSM in Germany that focussed on STIs and was conducted in 2006. For the biggest of three different study arms, participants were recruited online: German language MSM contact-, dating-, and chat-websites were asked to provide a link to the questionnaire website for their users. Of 19 websites approached, seven websites participated, including the largest German language websites. The mode of advertisement for participating in the KABaSTI study ranged from individual information to all website users by a newsletter, providing a prominent link on the start page, to providing a more hidden link on an information resources page. The referring website was recorded for evaluation. Postal code and country of residence data were collected in early questions, while HIV testing history and HIV status data were collected in the last part of the questionnaire. The drop-out between the first and the last questions was 35–40%. There were minor differences in regional distribution of participants who dropped out and those who completed the questionnaire. However, these minor differences did not result in any appreciable regional bias concerning distribution by federal state or by rural – metropolitan areas.

### Recruitment of the offline sample

Offline participants were recruited in 76 private medical practices, who since 2001 had reported syphilis infections in MSM to the German infectious disease surveillance system. A considerable proportion of these practices have specialized in HIV care. Most participating practices were located in larger cities. Since offline participants reflect the geographical distribution of HIV specialised practices rather than geographical distribution of MSM in Germany the offline sample of the study was excluded from this analysis.

The KABaSTI study was approved by the ethical committee of the Charité University Clinic in Berlin.

#### GMA-2007-study

The Gay Men and AIDS (GMA) survey of 2007 was the latest in a series of periodic behavioural surveys among MSM in Germany, started in 1987 [12]. In GMA-2007, participants were recruited online (basically like in the KABaSTI-study) and offline through nine magazines for gay men, that included the print questionnaire in their May edition. All magazines but one are available free of charge in gay venues in the larger cities. The questionnaire was self administered, and no incentives were offered for participation.

GMA participants were also asked whether they had participated in the KABaSTI study or other similar online surveys in 2006, and offline participants were asked whether they already had filled out an online questionnaire. Both surveys – KABaSTI and GMA – collected demographic data, including the first two digits of the five digit postal code of the place of residence, data on population size of the place of residence, educational status, sexual identity, HIV testing behaviour and HIV status [13,14].

Questionnaire data were analysed for consistency and validity (e.g. online questionnaires filled out in less than ten minutes were excluded). For statistical analysis we used SPSS, versions 14.0 and 15.0.

#### GayRomeo User data 2008

The regional distribution of participants of the above described surveys was compared with publicly available data on GayRomeo user regional distribution, which is provided on the website and is based on self-reported data from the user profiles. Data were accessed in January 2008 and refer to almost 290,000 currently active profiles of users living in Germany. We consider this the largest publicly available dataset on regional distribution of MSM in Germany.

### Data on the size and regional distribution of the general and MSM Population

For the size and distribution of the general population in postal code areas we used commercially available data provided by *Infas Geodaten *for the adult population aged 20–59 years dated from 2001. These data are not available stratified by sex. For this analysis it was assumed that men and women in this age group are equally distributed in the postal code areas. The difference of the total male adult population in the age group 20–59 years between 2001 and 2007 was 31,000 (0.14%) and was neglected. We assumed no significant change in the distribution by postal code areas between 2001 and 2006/07

Estimates of the proportion of MSM in the adult male population in Germany: the Federal Agency for Health Promotion (Bundeszentrale für gesundheitliche Aufklärung) collected data on sexual preference and experience in representative telephone surveys with 3,100 adult male participants in late 2007 for regular evaluation of HIV related health promotion activities. 2.5% (95%CI 1.5 – 3.4) of the male participants in the age group 20–50 years reported sexual contacts with men in the previous 12 months [[15], *BZgA, personal communication*]. Since sex between persons of the same gender is still a stigmatised behaviour, we believe that the upper range of the confidence interval provides a more realistic estimate than the lower range. Based on an estimated proportion of 2.5 to 3.4% and population statistics provided by the Federal statistics agency (Statistisches Bundesamt), we estimated the number of MSM in the adult population between 20 and 59 years of age in Germany at 575,000 to 785,000 persons. For calculation of regional MSM populations we used an estimate of 600,000.

### Comparison of study samples with another internet convenience sample and with national surveillance data

To evaluate the extent to which survey participants are representative for German MSM in terms of regional distribution, we compared the two study samples with each other and with the Gay Romeo user profiles. In addition, the proportional regional distribution of survey participants who reported an HIV diagnosis later than 1995 was compared with HIV surveillance data from 1996 through 2007 for regional distribution. For the analysis, HIV notifications within the surveillance system had to fulfil two conditions: (1) attributed to MSM by the notifying physician (2) the postal code of the residence place (PCA) was available for the patient. 11,657 notifications were included. Case reports with postal code available only from the health care provider (n = 3,956) or the laboratory (n = 2,132) were excluded. For the comparison of the proportional regional distribution between surveillance and survey data both survey samples were combined.

Details of the German infectious disease surveillance system for HIV have been described elsewhere [16].

## Results

### Study populations

For the KABaSTI-study we received 6,958 online questionnaires of which 1,600 (23%) were excluded. The most frequent reason for exclusion was residence outside of Germany (35%), followed by early termination of the online questionnaire after the first or second question (12%), fill-out times of less than ten minutes, highly implausible or contradictory responses, and reported age below 16 or above 85 years. Young persons and persons with low educational status were overrepresented among the excluded questionnaires.

For the GMA-2007-survey, 9.724 questionnaires were completed. Of those, 886 from participants residing in Austria were evaluated separately. 357 were excluded because no country of residence was reported. 311 questionnaires were excluded for other reasons (completion in too short time, no age specified, highly implausible answers, other countries of residence than Germany). Offline participants who reported participation in the internet survey were also excluded (n = 88). 8,170 remaining questionnaires could be evaluated, 1,975 of them were submitted offline and 6,195 online.

**Figure 1:**
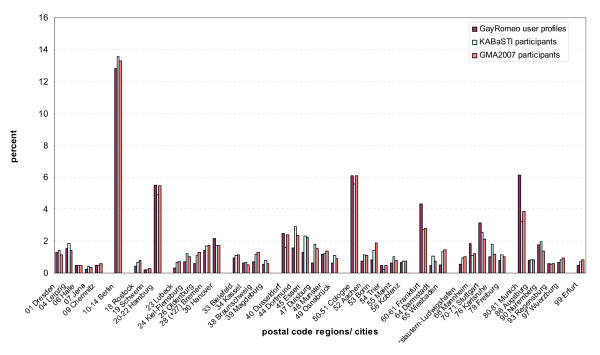
**Comparison of the postal code distribution of survey (KABaSTI + GMA-2007) participants and GR user profiles for selected postal code regions**. Bars represent the proportion (%) of survey participants/GR user profiles living in the respective postal code area.

When we compared the proportional geographical distribution of KABaSTI online participants with the proportional geographical distribution of all GMA-2007 participants and the online-only sample of the GMA-2007 survey with the distribution of the GayRomeo™ user profiles (see Figure 1), the match was better for the total GMA survey population. In the GMA online sample the proportion of participants from the largest gay centers (Berlin, Hamburg, Munich, Cologne, Frankfurt) was smaller. A large proportion of survey participants from these cities were recruited through gay magazines, and since we had used the two recruitment methods alternatively and had excluded online participants, who indicated offline participation, we feel it is justified to combine on- and offline samples of the GMA survey for the purpose of estimating the proportional regional distribution of MSM.

The websites which participated in the GMA-2007-survey partly overlapped with the websites participating in the KABaSTI-study (see Table 1). There was a moderate overlap between survey participants: 4% of the GMA-2007-survey participants in 2007 reported participation in the KABaSTI-study, and 21% had participated in an internet survey in 2006, but could not remember which one (beside KABaSTI there were three more large Internet surveys conducted in 2006 with a cumulated number of approximately 75,000 participants). 18% of the KABaSTI-participants and 8% of the GMA-participants were recruited from the GayRomeo website.

**Table 1:** Basic characteristics of KABaSTI and GMA-2007 survey participants

		KABaSTI (online) (2006) (n = 5,358) (%)	GMA-2007 (n = 8,170) (%)
**Age distribution**			
	15–20	11.3	11.2
		
	21–29	28.8	25.3
		
	30–44	44.8	40.1
		
	>44	15.0	23.4
		
**Educational status**	No qualification for university access	48.8	42.2
		
	Qualification for university access	51.2	57.8
		
**City size**			
		
	> 1 million	22.1	23.8
	500,000–1 million	12.1	13.3
	100,000–500,000	22.6	20.3
	20,000–100,000	22.0	21.1
	< 20,000	21.1	21.5
			
		
**Sexual identity**	Gay/homosexual	88.3	84.8
		
	bisexual	10.3	10.6
		
	Other/none	1.4	4.6
		
**HIV testing history**	Ever tested	69.0	64.3
		
	Never tested	31.0	35.7
		
**Self-reported HIV status among tested**	HIV positive	17.5	10.7
		
	HIV negative	80.6	89.3
		
	no test result specified	1.9	-
		
**Recruitment sites**			
		
online	Gay Romeo	18.1	8.1
		
	Gay Royal	15.3	30.4
		
	Homo.net	29.1	7.8
		
	Queer.de	12.3	9.5
		
	Funkyboys (young MSM)	13.7	12.6
		
	DBNA (young MSM)	-	6.5
		
	Bareback City (bareback)	9.2	-
		
	others	2.3	1.0
		
offline	Gay print media	-	24.1

The composition of the online samples differed according to the websites from which the participants were linked to the questionnaire.

The most obvious differences existed for participants recruited on a website for young MSM (younger, more often living outside of larger cities, lower educational status, higher proportion of lower paid jobs), and for participants recruited on a bareback website (older, highest proportion of participants with HIV or previous STIs, a high proportion living in metropolitan areas, highest proportion of unemployed participants).

Comparing all online participants of the KABaSTI-study with all GMA-2007 participants, no relevant differences were found with respect to age, educational or professional status, population size of the place of residence, sexual identity, and HIV testing history (see Table 1). A significantly higher proportion of KABaSTI participants reported to be HIV positive. This can be explained by one bareback website in the KABaSTI study, which contributed almost 50% of all HIV positive KABaSTI participants. Excluding these participants, the proportion of HIV positive participants were virtually the same in both surveys.

In comparison with the general population, the study survey populations were younger (men aged 50 years or more are strongly underrepresented), better educated (the lowest educational degree achieved by 51% in the general and 14% (KABaSTI) resp. 13% (GMA) in the study population, highest degree achieved by 22% in the general and 51% (KABaSTI) resp. 58% (GMA) in the study population and had a higher professional status. The unemployment rate was comparable with the general population.

### Comparison of the regional distribution of survey participants and GayRomeo user profiles

Regional allocation of survey participants was based on the first two digits of the five digit postal code, while user profile distribution is based on cities. Most large cities and the 16 federal states of Germany can be matched with reasonable reliability with two digit postal code areas (PCA). With few exceptions (PCA 14: Berlin – Brandenburg, PCA 21: Hamburg – Lower Saxony, PCA 22 Hamburg – Schleswig-Holstein, PCA 27 Bremen -Lower Saxony) state overlapping postal code areas are rural areas with low population density. For the comparison of regional distribution between surveys and website profiles (Figure 1, Table 2) the mentioned state-overlapping PCA were attributed to the cities, i.e. PCA 14 to Berlin, PCA 21 and 22 to Hamburg, and PCA 27 to Bremen. A comparison of the proportional distribution of survey participants and user profiles for large cities in Germany and the respective postal code areas is shown in Figure 1. Many of the smaller differences between survey participants and user profiles are likely due to the different data formats: Higher proportions of web profiles than survey participants can be found in a few larger cities (Frankfurt, Hanover, Munich). This may – at least partly – reflect men living in the surrounding areas of these cities, while lower proportions in many medium sized cities and especially in the densely populated Rhine-Ruhr region (e.g. Jena, Erfurt, Braunschweig, Oldenburg, Dortmund, Essen, Duisburg) reflect the larger areas defined by the postal code regions, which cover more than just the largest city in the respective postal code area.

**Table 2:** Estimates* for the regional population size of MSM in Federal States and largest cities in Germany

Source for estimate	KABaSTI (online) 2006	GMA-2007	GayRomeo User Profiles 2008
German Federal States, in alphabetical order (postal code regions)	MSM total (20–60 y)		

Baden-Wuerttemberg (68 – 79, 88, 89)	69,000	62,000	71,000

Stuttgart (70, 71)	15,000	13,000	19,000

Bavaria (80 – 87, 90–97)	64,000	66,000	85,000

Munich (80 – 81)	19,000	23,000	37,000

Berlin (10 – 14)	81,000	80,000	77,000

Brandenburg (14 – 17)	8,000	7,000	7,000

Bremen (27, 28)	10,000	10,000	9,000

Hamburg (20 – 22)	29.000	33.000	33.000

Hesse (34–36, 60 – 65)	50,000	49,000	48,000

Frankfurt (60 – 61)	16,000	17,000	26,000

Mecklenburg-Vorpommern (17 – 19)	8,000	9,000	8,000

Lower Saxony (26,29–31, 37,38,49)	42,000	44,000	39,000

Hanover (30)	10,000	10,000	13,000

North Rhine- Westphalia (32, 33, 40–48, 50 – 53)	150,000	159,000	139,000

Cologne (50,51)	34,000	37,000	37,000

Duesseldorf (40)	10,000	14,000	15,000

Rhineland-Palatina (54 – 58, 67)	17,000	18,000	19,000

Saarland (66)	8,000	7,000	8,000

Saxony (01–04, 08,09)	29,000	25,000	25,000

Leipzig (04)	11,000	9,000	9,000

Saxony-Anhalt (39, 06)	11,000*	10,000	10,000*

Schleswig-Holstein (22–25)	14,000	14,000^&^	11,000^&^

Thuringia (07, 98,99)	9,000	10,000	10,000

*Assumption concerning total size of the German MSM population in age groups 20–59: 2.6% of adult male population = 600,000; estimates rounded to 1,000

We calculated MSM population sizes of federal states and the largest cities of Germany based on the proportional regional distribution of survey participants and user profiles, assuming a total population size of 600,000 for MSM in the age range between 20 and 59 years (Table 2), which represents 2.6% of the total male population in these age groups in Germany. With few exceptions the ranges of the estimates for the 16 federal states calculated from the three different data sources were small. Noteworthy are the larger discrepancies between survey-based estimates und user profile based estimates in Bavaria and North-Rhine Westfalia. (see Table 2).

### Comparison of the relative regional distribution of prevalent HIV infection in internet survey participants and national HIV surveillance data

**Figure 2:**
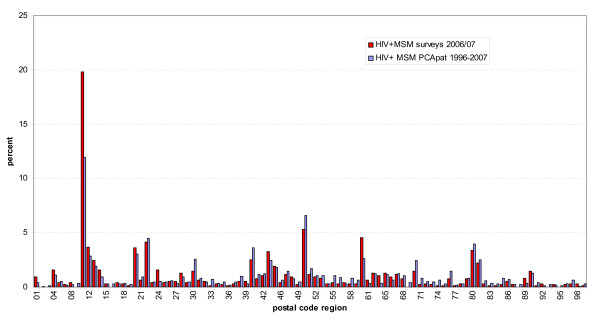
**Comparison of proportional regional distribution of self-reported HIV infections (n = 948) in KABaSTI and GMA survey participants with proportional regional distribution of newly diagnosed HIV infections in MSM (n = 11,657 cumulative from 1996–2007) in the national surveillance system**. Bars represent the proportion (%) of HIV positive survey participants and the proportion of newly diagnosed HIV infections in MSM from 1996–2007 in the national surveillance system. Selected postal code areas: 04 = Leipzig; 10–14 = Berlin; 20 – 22 = Hamburg; 28 = Bremen; 30 = Hanover; 40 = Duesseldorf; 44 = Dortmund; 45 = Essen; 47 = Duisburg; 50 = Cologne; 60 = Frankfurt; 70 = Stuttgart; 80–81 = Munich.

A comparison between the regional distribution of participants who report to be HIV positive in the KABaSTI and GMA surveys (n = 948) and newly diagnosed HIV in MSM (1996 through 2007) from the regular infectious disease surveillance system (based on n = 11,657 patients) revealed similarities in the crude distribution with some remarkable differences for Berlin, Frankfurt, Hanover and Stuttgart (see Figure 2).

### Estimating regional MSM concentration factors

**Figure 3:**
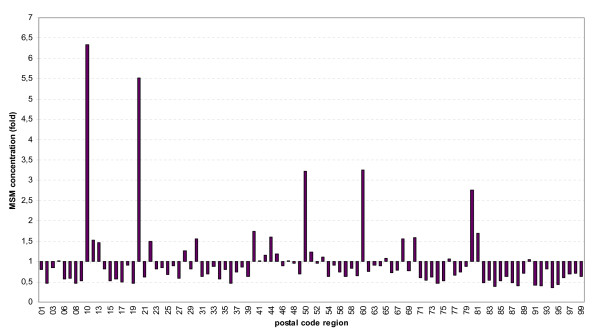
**Relative regional distribution of survey participants (MSM) in relation to relative distribution of 20–59 year old males from the general population of Germany**.

Based on these comparisons, we suggest that the regional distribution of MSM participating in the internet surveys roughly reflects the regional distribution of the German MSM population as a whole. If the proportion of participating MSM in each of the 95 German postal code regions (defined by the first two digits of the five digit postal codes) is divided by the proportion of the general adult population residing in the respective regions, the resulting value describes the concentration of MSM in the respective areas ("MSM concentration factor": essentially an odds ratio for study participation in relation to the general population). The result for both surveys combined is shown in Figure 3. The highest concentrations of MSM can be found in the inner cities of Berlin (6-fold), Hamburg (4–6-fold), Frankfurt (3-fold), Cologne (3-fold), and Munich (2.5–3-fold)(1-fold = proportion of MSM living in the respective postal code region is equal to the proportion of the general adult male population). Also in other larger cities like Hannover, Bremen, Stuttgart, Mannheim, Essen, Duesseldorf and Dortmund, the proportion of MSM was elevated. These MSM concentrations in the largest German cities are counterbalanced by a lower concentration (concentration factor < 1.0) of MSM in many rural regions, especially in southern, eastern and central parts of the country. If the regional distribution of MSM is stratified by age, the concentration factors in the larger cities disappear for the age group of MSM younger than 26 years (data not shown). Also stratification by educational status has a significant impact on the size of the concentration factor in large cities: MSM with higher educational status are more likely to migrate to larger cities.

## Discussion

### Potential Bias in the offline sample of the GMA survey

Because the GMA-offline sample was recruited by gay magazines which are primarily distributed in gay venues of the largest cities, this sample may be slightly biased towards men older than 25–30 years living in metropolitan cities. However, while the KABaSTI-offline sample represents patients (with a high probability of being HIV-positive or having a history of previous STIs) and the geographical location of practices, the GMA-offline sample represents middle class middle age gay men in larger cities and the respective catchment areas. Because of these differences, we feel it was justified to exclude the offline sample of the KABaSTI-study while we included the offline sample of the GAM survey for the estimation of the regional distribution of MSM.

### Representativeness of Internet surveys for regional MSM distribution

High precision in estimating MSM population sizes is an elusive goal due to the lack of a precise definition of MSM and measurement problems. We demonstrate that in a post-industrialized country like Germany, quite representative samples of sexually active middle-class MSM can be reached with web-based surveys. Participation biases for MSM subpopulations are likely to be much smaller for internet convenience samples of MSM than for MSM convenience samples approached in any other setting; however biases probably still remain for MSM who do not self-define as gay men, older MSM (> 50 years of age), MSM from migrant communities, and MSM with lower educational or professional status. Web-recruited MSM samples are also likely to be more sexually active than MSM in a probability sample of the total population, which is true also for other gay venue based samples. We can detect a significant impact of educational status on the population size of the place of residence, and it has been described that men with a lower educational status less often self-identify as gay [17] and seem to participate in Internet surveys to a lower degree than MSM with a higher educational status. Thus it may be that the regional distribution of lower class MSM who are reached less well by Internet surveys or are less willing to participate in such surveys differs from our sample.

For the higher population size estimates based on user profiles compared with survey participants for most of the larger cities there may be several interpretations. The most likely explanation is that the regional allocation in the user profiles is not as much based on the real place of residence, but rather on the "MSM catchment area" of the respective cities. This would result in inflated estimates for large cities in densely populated areas (e.g. Munich, Frankfurt, Hanover and Stuttgart), while estimates for cities with less densely populated surroundings (e.g. Berlin, Leipzig) would be less variable (see Table 2). For Hamburg, Bremen and Cologne we reduced this "catchment effect" already by including postal code areas which only partly cover city areas.

Other possible reasons might be that MSM from metropolitan areas are less willing to participate in behavioural surveys than MSM outside these areas, or that a higher proportion of metropolitan MSM is visiting MSM websites, i.e. that the penetration of MSM websites in the urban MSM population is higher, while the proportion of MSM from different areas willing to participate in behavioural surveys is similar or equal. Thus, it remains an unresolved question, whether MSM population estimates for some of the largest cities derived from survey participant distribution reflect reality more or less precise than estimates derived from the user profile distribution.

Another potential bias can be regional differences in the preferred websites for MSM, which we tried to minimize by including a mix of websites for the recruitment of survey participants. However, we had no influence on the method of advertising the surveys. The website with the largest user number had decided to promote the surveys only by a relatively hidden link on an information resource page, while other websites with a more skewed regional popularity had promoted the surveys more prominently. As we can show by comparing the survey participant distribution between participants recruited on Gay Romeo and the other websites, this may have contributed to some bias in regional participation rates which may explain the discrepancies in the estimates for Bavaria and North Rhine-Westphalia derived from the surveys and the user profiles: From the KABaSTI participants who were recruited on GayRomeo 14.6% reported residence in Bavaria, 19.3% residence in North Rhine-Westphalia, compared with 10.6% and 25% of all participants. Similarly, by inclusion of a regionally popular website in the KABaSTI study, the KABaSTI-based estimate for Saxony and Saxony-Anhalt may be slightly inflated (see Table 2).

While we found little published literature on geographical representativeness of internet convenience samples of MSM, Evans et al., who compared an internet convenience sample of MSM from Great Britain with a national probability sample [18], also found a similar geographical distribution of MSM in both samples.

The comparison between the regional distribution of diagnosed HIV infections in KABaSTI participants and the regional distribution of diagnosed infections reported in the national surveillance system (Fig. 2) reveals some minor and some more pronounced differences. To minimise biases due to the low absolute numbers of diagnosed infections in the internet survey participants we combined both surveys for this analysis. Differences therefore exist in regions with low numbers of internet survey participants, or low numbers of reported infections. But also for several larger cities discrepancies exist: in Munich, Cologne, Duesseldorf, Stuttgart and Hanover (PCA 80, 50, 40, 70, 30) the proportion of HIV reports in the surveillance data is higher than the proportion of survey participants, while in Berlin and Frankfurt (PCA 10–14, PCA 60–61) it is the other way round. We believe that for Munich, Cologne, and Duesseldorf this may still be partly due to medical tourism from the surrounding areas, although we tried to reduce this confounder by restricting the analysis to cases reported with the PCA of the patient's place of residence. However, there may still be some level of misreporting the PCA of the health care provider as the PCA of the patient. This interpretation is supported by the observation that when we use the distribution of surveillance reports for comparison, where the PCA refers to both the place of residence of the patient and of the care provider, the differences become larger. For Stuttgart and Hanover however there is no difference in this regard. For these cities internal migration to other cities seems the most plausible explanation to us. Berlin would be expected to be the most attractive destination city for such internal migration movements because of its large gay community and well developed medical and subculture infrastructure. Also Frankfurt may be a destination city for the same reasons.

Another factor that could contribute some bias would be regional differences in internet access. However, after 2003, there are no major regional gaps in internet access, and availability of high speed or broad band internet connections in Germany is highly common.

## Conclusion

To summarize, the spatial distribution of participants of internet convenience samples may be used as a tool to estimate the regional distribution of a "hidden" population like MSM. Potential biases should be considered, which may arise from subtle differences in regional participation rates and recruitment on websites with skewed user characteristics. Compared to infectious disease surveillance data, self reported infection status data from surveys have the potential to detect post-HIV diagnosis migration patterns.

## Competing interests

The authors declare that they have no competing interests.

## Authors' contributions

MB conceived and designed the GMA studies, UM conceived and designed the KABaSTI study. UM, MB and AJS designed the survey questionnaires. The KABaSTI and GMA survey were coordinated by AJS, data analysis and interpretation were done by UM, AJS, and MB. OH contributed to conception and questionnaire of the KABaSTI study. The idea for linking and comparing regional distribution of survey participants with statutory surveillance data came from UM, AJS compared the KABaSTI sample with the GMA sample. The manuscript was drafted by UM and AJS and critically revised by OH and MB. All authors read and approved the final manuscript.

## Pre-publication history

The pre-publication history for this paper can be accessed here:


